# Mosaic STAT5B gain-of-function associated with demyelinating disease and autoimmunity

**DOI:** 10.70962/jhi.20250060

**Published:** 2025-07-08

**Authors:** Erica G. Schmitt, Nermina Saucier, Samuel I. Risma, Sena N. Arbag, Ana Kolicheski, Alexander J. Paul, Tomi L. Toler, Katarina Semkiu, Soe S. Mar, Joshua D. Milner, Jeffrey J. Bednarski, Megan A. Cooper

**Affiliations:** 1 Department of Pediatrics, Division of Rheumatology and Immunology, Washington University School of Medicine, St. Louis, MO, USA; 2 Department of Pathology and Immunology, Washington University School of Medicine, St. Louis, MO, USA; 3 Department of Pediatrics, Westchester Medical Center, Valhalla, NY, USA; 4 Department of Pediatrics, Division of Genetics and Genomic Medicine, Washington University School of Medicine, St. Louis, MO, USA; 5 Department of Pediatrics, Division of Hematology and Oncology, Washington University School of Medicine, St. Louis, MO, USA; 6 Department of Neurology, Washington University School of Medicine, St. Louis, MO, USA; 7 Division of Pediatric Allergy, Immunology and Rheumatology, New York-Presbyterian/Columbia University Irving Medical Center, New York, NY, USA

## Abstract

Mosaic STAT5B gain-of-function was identified in a patient with adrenal insufficiency and significant neurological disease. These findings broaden the clinical spectrum of STAT5B defects, highlight the importance of sampling different tissues, and provide evidence for curative hematopoietic cell transplantation.

## Teaser

A patient with a predominantly neurological phenotype was found to have a mosaic STAT5B gain-of-function variant and was successfully treated with HCT.

The number of known monogenic inborn errors of immunity (IEI) is rapidly growing. The Janus kinase (JAK) and signal transducer and activator of transcription (STAT) signaling pathways are highly conserved and involved in a variety of cellular processes, and deficiency or dysregulation of JAK/STAT pathways is implicated in IEI, autoimmunity, and malignancy. Somatic mosaicism is an increasingly recognized genetic mechanism of IEI (1). Somatic gain-of-function (GOF) variants in *STAT5B* drive leukemias and lymphomas, and variants in the Src homology 2 (SH2) domain were reported in three patients with nonclonal eosinophilia, atopic dermatitis, diarrhea, and urticaria (2, 3, 4, 5). Here, we describe a previously healthy, developmentally normal 7-year-old Middle Eastern female presenting with autoimmune adrenal insufficiency and subacute lower extremity weakness with combined central and peripheral nervous system demyelination found to have a pathogenic somatic *STAT5B* variant. Written and informed consent was obtained for all participants, and studies were approved by the Institutional Review Board at Washington University.

**Figure 1.**
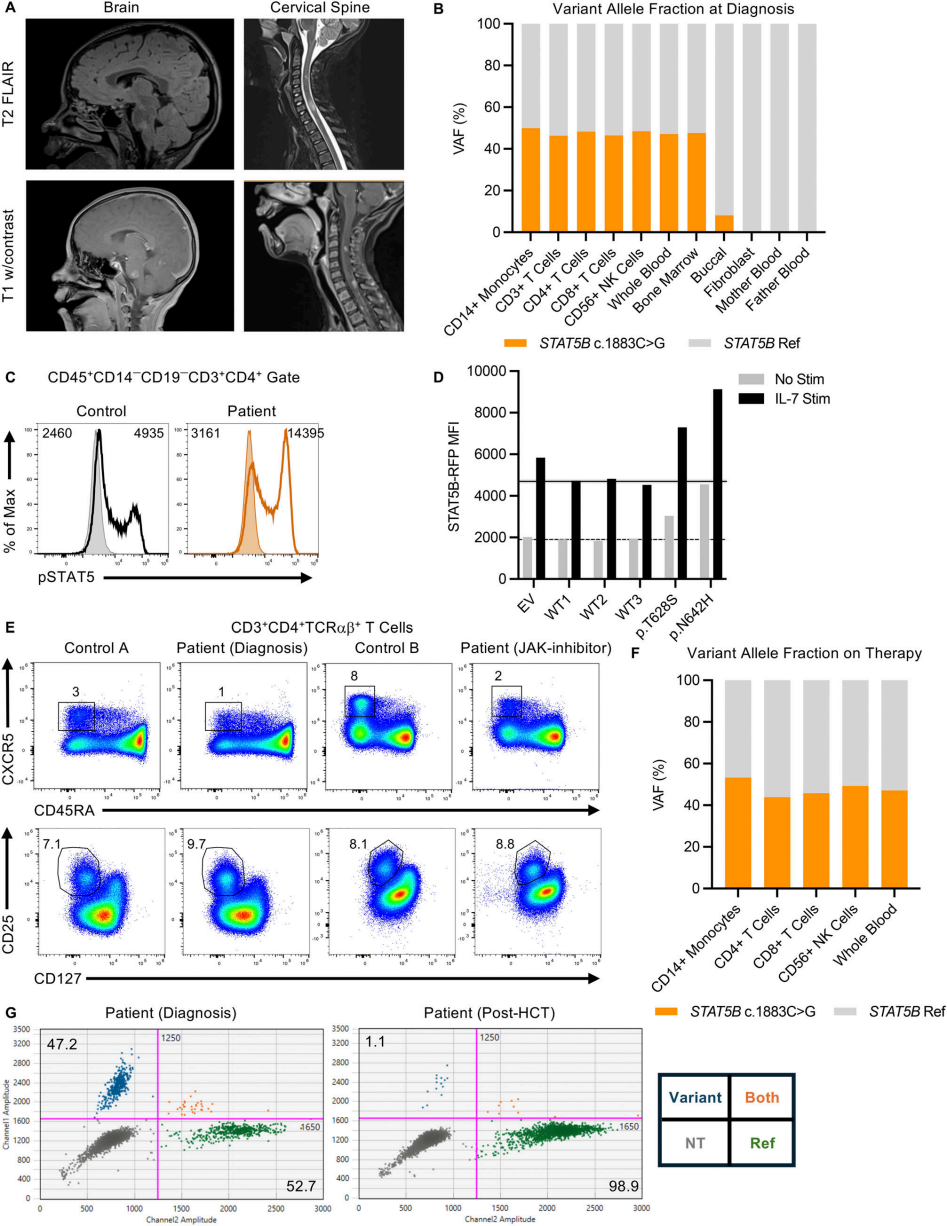
**Clinical, genetic, and functional analysis of a mosaic *STAT5B* variant. (A)** Multifocal, enhancing lesions with increased T2 fluid-attenuated inversion recovery (T2/FLAIR) hyperintensity in the brain at diagnosis in the pons and cervical spine. **(B)** ddPCR at diagnosis of *STAT5B* reference and *STAT5B* c.1883C>G VAFs in patient and parental DNA isolated from indicated tissues or FACS-sorted immune cells (T cells [CD3^+^], CD4 T cells [CD3^+^TCRαβ^+^CD4^+^], CD8 T cells [CD3^+^TCRαβ^+^CD8^+^], B cells [CD3^−^CD19^+^], monocytes [CD3^−^CD14^+^], natural killer [NK] cells [CD3^−^CD56^+^]). ddPCR (Bio-Rad) was performed with DNA from the indicated tissues with probes specific for the *STAT5B* c.1883C>G variant (FAM dye, custom design, Bio-Rad) and analyzed with the QX200 droplet reader and QX Manager software. Data represent two technical replicates and two separate buccal samples. **(C)** Patient and control peripheral blood mononuclear cells were stimulated with IL-2 for 20 min (Chiron, Proleukin) and pSTAT5 (pY694, Cat# 612599; BD Biosciences) assessed by flow cytometry (median fluorescence intensity, MFI) shown as unstimulated (shaded) versus stimulated (solid line) gated on live, CD45^+^CD19^−^CD14^−^CD3^+^CD8^−^CD4^+^ T cells. Assay was performed while the patient was off steroids. **(D)** STAT5B-RFP MFI measured in a reporter T cell line transfected with a GFP-tagged EV, or plasmids with WT *STAT5B* or the indicated *STAT5B* variants. RFP MFI was assessed in GFP^+^ cells that were unstimulated or stimulated with IL-7 for 24 h. Mean ± SEM of unstimulated and stimulated WT plasmids are indicated by the horizontal dashed lines and shading. The STAT5B reporter T cell line was established by transducing T28 cells with a STAT5B-RFP reporter lentivirus. The STAT5B reporter T cell line was then transfected with GFP-tagged STAT5B plasmids as indicated. Transfected cells were stimulated with IL-7 for 24 h, and RFP was measured by flow cytometry. Data shown are representative of three independent experiments. **(E)** Representative flow cytometry showing reduced CD3^+^CD4^+^ TCRαβ^+^ CXCR5^+^CD45RA^−^ cTfh cells (top row) and frequency of CD3^+^CD4^+^ TCRαβ^+^ CD25^+^CD127^−^ Treg cells (bottom row) in the patient compared with age-matched healthy controls at diagnosis and after 6 mo of tofacitinib. cTfh cells and Treg cells were pregated as CD45^+^ CD19^−^ CD14^−^ CD56^−^ TCRγδ^−^ CD3^+^ CD8^−^. **(F)** ddPCR for *STAT5B* c.1883C>G variant after 6 mo of therapy with tofacitinib in patient DNA isolated from whole blood or sorted cell populations. **(G)** ddPCR of patient peripheral blood DNA at diagnosis (left) and 20 mo after HCT (right) showing percentage of droplets with *STAT5B* c.1883C>G variant (Var) or WT *STAT5B* reference sequence (Ref). Data are representative of two technical replicates. NT, no template. Both, both templates. ddPCR, droplet digital polymerase chain reaction; EV, empty vector.

Upon presentation, the patient’s clinical exam was notable for ataxia, absent patellar and Achilles reflexes bilaterally, reduced vibratory sensation, and weak foot dorsiflexion. Magnetic resonance imaging (MRI) of the brain and spinal cord revealed central nervous system demyelination with multifocal, enhancing white matter lesions of the left frontal lobe, pons, cerebellum, and spinal cord (Fig. 1 A). Electromyography and nerve conduction velocity were consistent with a demyelinating polyneuropathy of motor nerves. Computed tomography of the chest, abdomen, and pelvis identified bilateral <5-mm pulmonary nodules and hilar lymphadenopathy. Complete blood count was notable for leukocytosis (peak WBC 22,000 cells/cumm; reference 4.5–13.5 K/cumm) with eosinophilia (peak 3,100 cells/cumm; reference 0.1–1.6 K/cumm). Immunophenotyping demonstrated normal IgG, IgG subclasses, and IgA, elevated IgM (233.1 mg/dl; reference 48–207 mg/dl), and mildly elevated IgE (135 IU/ml; reference 3.1–110.8 IU/ml). She had T cell lymphocytosis with peak CD4 5,165 cells/mcL (reference 650–1,500 cells/mcL) and CD8 2,249 cells/mcL (reference 370–1,100 cells/mcL). Cerebral spinal fluid (CSF) studies showed elevated protein, nucleated cells, IgG and IgG synthesis rate, and albumin, with increased oligoclonal bands (three bands). Extensive infectious, metabolic, and autoantibody testing was unremarkable, aside from serum anti-MOG titer positive at 1:20 (normal <1:20). Serum anti-MOG titer repeated 1 mo later was negative. Flow cytometry and cytology from CSF and blood were not concerning for malignancy. She received high-dose methylprednisolone followed by corticosteroid taper and intravenous immunoglobulin (IVIG) 1 g/kg monthly. Follow-up imaging 5 mo later showed an enlarged thymus thought to represent rebound thymic hyperplasia and unchanged pulmonary nodules. At 7 mo, she had subacute worsening and severe sensory ataxia, with ultrasound demonstrating mild splenomegaly and positron emission tomography–computed tomography with homogeneous moderate uptake in the thymus and scattered F-18 fluorodeoxyglucose (FDG) activity in the cervical and thoracic spinal cord, but no other suspicious nodal or extranodal uptake. MRI brain and spine showed increased short tau inversion recovery (T2/STIR) hyperintense and enhancing lesions in the thoracolumbar spine. She received two doses of rituximab (750 mg/m^2^), high-dose methylprednisolone, and remained on a prolonged steroid taper and monthly IVIG.

Clinical panel genetic testing was performed on a peripheral blood sample and identified two heterozygous *STAT5B* variants. The first was a missense change (c.1883C>G, p.Thr628Ser) at position Chr17:42210194 (GRCh38) heterozygous in two individuals in gnomAD (v4.1.0), with a Combined Annotation Dependent Depletion (CADD) (v1.7) score of 24.7. The second variant was a missense change (c.2161G>A, p.Gly721Ser) at position Chr17:42202416 present in 156 individuals, including 2 homozygous (both of Middle Eastern ancestry) in gnomAD, with a CADD score of 4.3. She had a pathogenic heterozygous variant in *LIPA* and heterozygous variants of uncertain significance in genes inconsistent with her clinical phenotype including *ADAM17*, *AK2*, *COPA*, *LIG4*, *POLR3A*, *PRKDC*, *RELA*, *SIAE*, *SLC7A7*, and *TNFAIP3*, all of which were subsequently determined by clinical trio whole-exome sequencing (WES) to be inherited from an unaffected parent. Interestingly, the *STAT5B* p.Thr628Ser variant was not initially reported on clinical WES, which was performed with DNA isolated from a buccal sample. However, on reanalysis it was present in 13 of 111 (∼12%) sequencing reads, suggesting mosaicism, and absent from parental samples. The *STAT5B* p.Gly721Ser variant was inherited from the proband’s healthy mother, who was homozygous for this variant, and is thus considered benign. No other pathogenic variants were identified by trio exome sequencing.

The *STAT5B* p.Thr628Ser variant, located in the SH2 domain, has been reported in T cell prolymphocytic leukemia (2). Functional studies have demonstrated that the variant confers GOF, as shown by assays in cell lines that express the variant, which exhibited constitutive hyperphosphorylation, increased STAT5B transcriptional activity, and cytokine-independent cell growth (2). Additional clinical targeted Sanger sequencing of the p.Thr628Ser variant again detected apparent mosaicism in buccal swab DNA and ∼50% frequency in blood and bone marrow. Repeat peripheral blood analysis and bone marrow aspirate were normal, showing normocellular bone marrow with progressive multilineage hematopoiesis without clonal B cell or aberrant T cell populations, and normal fluorescence in situ hybridization for myelodysplastic syndrome.

We utilized a droplet digital PCR assay to determine the specific variant allele fraction (VAF) in different tissues. This demonstrated a VAF ∼8% in buccal DNA, and apparent heterozygosity in DNA from whole blood (47%) and bone marrow (48%) (Fig. 1 B). Purified immune cell subsets did not identify enrichment in tested cell types (Fig. 1 B). The patient had received rituximab, and B cells could not be assessed. The variant was absent from a skin punch biopsy–derived fibroblast line, suggesting somatic mosaicism limited to the hematopoietic compartment (Fig. 1 B). Deep sequencing of peripheral blood DNA using a custom-targeted research gene panel, including 71 genes associated with immune dysregulation, did not identify any additional germline or mosaic variants (gene list available upon request).

Analysis of phosphorylated STAT5 (pSTAT5) expression in patient T cells after cytokine stimulation showed increased responsiveness (Fig. 1 C). A STAT5B-RFP transcriptional reporter T cell line was established, and increased RFP expression was observed in a known GOF variant, *STAT5B* p.Asn642His (Fig. 1 D). RFP expression was also increased in the *STAT5B* p.Thr628Ser variant compared with WT controls, again confirming that this patient’s variant confers GOF (Fig. 1 D). Reduced circulating T follicular helper (cTfh) cells and increased regulatory T (Treg) cells have been described in patients with somatic STAT5B GOF, likely due to overactive STAT5B signaling (3). The patient displayed a reduction in circulating cTfh cells and similar or slightly increased Treg cells compared with healthy controls (Fig. 1 E). JAK inhibition has been previously described as a treatment for patients with nonclonal STAT5B GOF, with a positive, though variable, response (5). Our patient received a second course of rituximab ∼1 year after presentation when her disease flared upon discontinuing steroids. After 3 mo, due to continued disease activity, she started tofacitinib, a JAK inhibitor primarily targeting JAK1/JAK3, initially 5 mg and then 10 mg twice daily. She improved clinically, including tolerating a lower dose of steroids, but was not able to discontinue steroids without disease flare. IVIG was discontinued but ultimately restarted at replacement dosing due to low IgG (379 mg/dl, reference 608–1,572 mg/dl). IgM was also low at that time (25.6 mg/dl, reference 52–353 mg/dl), and IgA was normal. The VAF of the *STAT5B* p.Thr628Ser variant in peripheral blood was stable at ∼47% after ∼6 mo of treatment with tofacitinib (Fig. 1 F). The VAF was between ∼44% and 53% in the lymphocyte populations that were assessed and was stable in most cell lines but did increase slightly in CD14^+^ monocytes (Fig. 1 F).

Based on her refractory disease, she underwent definitive therapy with haploidentical hematopoietic cell transplant (HCT) 2 years after presentation. She received reduced intensity conditioning (NCT03128996) with alemtuzumab (45 mg total on days −22 to −20), fludarabine (30 mg/m^2^/day on days −8 to −4), thiotepa (8 mg/kg on day −4), and melphalan (140 mg/m^2^ on day −3) followed by infusion of bone marrow hematopoietic cell product from her father (infused on day 0). Graft-versus-host disease (GVHD) prophylaxis included posttransplant cyclophosphamide (50 mg/kg/day on days +3 and +4), tacrolimus, mycophenolate, and abatacept (continued through 1 year after HCT). She achieved complete donor engraftment in all lineages (>95% donor) at day +30 after HCT and had no major infectious complications after transplant. She developed chronic GVHD of the skin at 7 mo after HCT that was treated with tacrolimus, ruxolitinib, and etanercept. After HCT, she had significant improvement in her neurological manifestations, with return of reflexes, improved strength, vibratory sensation, and gait. Chimerism monitoring at 18 mo after transplant demonstrated full donor engraftment for CD3^+^ T cells and CD15^+^ myeloid cells, with <5% recipient cells present below the limit of quantification. Peripheral blood obtained 20 mo after transplant detected the *STAT5B* p.Thr628Ser variant at a VAF of ∼1% (Fig. 1 G). VAF will continue to be monitored over time.

In summary, we present a case of mosaic STAT5B GOF precipitated by an early somatic event and limited to the hematopoietic compartment, thus prompting curative therapy with HCT. This case highlights features previously described in patients with mosaic STAT5B GOF, including immune dysregulation and eosinophilia, but expands the clinical spectrum to include significant neurological disease. Here, careful investigation of *STAT5B* variants in different tissues prompted the final diagnosis. The clinical spectrum of rare IEI is expanding and evolving, and genetic mosaicism remains an under-recognized disease mechanism that should be considered when screening patients for IEI.

## Data Availability

The data underlying Fig. 1 are available in the published article and from the corresponding author upon request.
